# Gut Microbial Protein Expression in Response to Dietary Patterns in a Controlled Feeding Study: A Metaproteomic Approach

**DOI:** 10.3390/microorganisms8030379

**Published:** 2020-03-07

**Authors:** Sheng Pan, Meredith A. J. Hullar, Lisa A. Lai, Hong Peng, Damon H. May, William S. Noble, Daniel Raftery, Sandi L. Navarro, Marian L. Neuhouser, Paul D. Lampe, Johanna W. Lampe, Ru Chen

**Affiliations:** 1 Institute of Molecular Medicine, the University of Texas Health Science Center at Houston, Houston, TX 77030, USA; sheng.pan@uth.tmc.edu (S.P.); hongpeng12@gmail.com (H.P.); 2 Fred Hutchinson Cancer Research Center, Division of Public Health Sciences, Seattle, WA 98109, USA; draftery@uw.edu (D.R.); snavarro@fredhutch.org (S.L.N.); mneuhous@fredhutch.org (M.L.N.); plampe@Fredhutch.org (P.D.L.); jlampe@fredhutch.org (J.W.L.); 3 Department of Medicine, University of Washington, Seattle, WA 98105, USA; llai@medicine.washington.edu; 4 Department of Genome Sciences, University of Washington, Seattle, WA 98105, USA; damon@gurple.com (D.H.M.);; 5 Department of Anesthesiology and Pain Medicine, Northwest Metabolomics Research Center, University of Washington, Seattle, WA 98109 USA; 6 Division of Gastroenterology and Hepatology, Department of Medicine, Baylor College of Medicine, Houston, TX 77030, USA

**Keywords:** Microbiome, proteomics, dietary intervention, SCFA, fatty acid metabolism, dysbiosis, mucin-degrading enzymes

## Abstract

Although the gut microbiome has been associated with dietary patterns linked to health, microbial metabolism is not well characterized. This ancillary study was a proof of principle analysis for a novel application of metaproteomics to study microbial protein expression in a controlled dietary intervention. We measured the response of the microbiome to diet in a randomized crossover dietary intervention of a whole-grain, low glycemic load diet (WG) and a refined-grain, high glycemic load diet (RG). Total proteins in stools from 9 participants at the end of each diet period (*n* = 18) were analyzed by LC MS/MS and proteins were identified using the Human Microbiome Project (HMP) human gut microbiome database and UniProt human protein databases. T-tests, controlling for false discovery rate (FDR) <10%, were used to compare the Gene Ontology (GO) biological processes and bacterial enzymes between the two interventions. Using shotgun proteomics, more than 53,000 unique peptides were identified including microbial (89%) and human peptides (11%). Forty-eight bacterial enzymes were statistically different between the diets, including those implicated in SCFA production and degradation of fatty acids. Enzymes associated with degradation of human mucin were significantly enriched in the RG diet. These results illustrate that the metaproteomic approach is a valuable tool to study the microbial metabolism of diets that may influence host health.

## 1. Introduction

Diet and the microbiome impact health, in part, through the microbial metabolism of dietary components. Anaerobic fermentation of dietary fiber produces short chain fatty acids (SCFA) which are important in gut homeostasis and have been strongly associated with host metabolic function [1]. Dietary fiber includes a large variety of carbohydrate polymers and refined and whole grain diets differ in their proportion and composition [2]. Diets enriched with refined grains have been associated with lower microbial diversity compared to a diet high in less refined whole grains [3]. There is metabolic specialization within the gut microbiome for a vast array of complex dietary polysaccharides. The microbiota produces a broad spectrum of enzymes that catalyze the depolymerization, degradation, and uptake of polysaccharides [4]. The interaction between the dietary polysaccharides of differing structural properties in conjunction with the suite of bacterial enzymes that degrade them dictates the host exposure to microbial metabolites [5,6].

Metaproteomics, the measurement of the bacterial proteins in the microbial community, provides a direct measure of gut bacterial activity and metabolism. Complementary to metagenomic and metatranscriptomic methods, metaproteomic analysis of the bacterial proteomes in the microbial community provides a more direct measure of functional status associated with gut bacterial activity and metabolism by globally characterizing proteins and corresponding microbial species in complex and diverse microbiomes [7]. A recent study which applied metaproteomics combined with 16S rRNA gene sequencing and metabolomics to investigate the effect of dietary resistant starch on human gut microbiome identified >50,000 proteins in the fecal samples of the study cohort [1]. Another study using a metaproteomics approach to study diet induced changes in mouse gut microbiome identified >11,000 protein groups and 167 KEGG pathways in the mouse fecal samples [2]. Unlike metagenomics (all of the genes and genomes in the microbial community), which measures the functional potential of the microbial community, metaproteomic signatures provide a direct measure of the microbial activity of the microbial community. We present here an integrated shotgun proteomic approach to measure the metaproteomes in fecal specimens. We applied this approach to characterize the response of the microbial metaproteome to controlled refined grain, high glycemic load diets versus whole grain, low glycemic load diets.

## 2. Materials and Methods 

### 2.1. Study Design 

Data and biologic samples were derived from the Carbohydrates And Related Biomarkers (CARB) study, conducted between June 2006 and July 2009 at the Fred Hutchinson Cancer Research Center (Fred Hutch, Seattle, WA, USA). The CARB study was a randomized, controlled, crossover feeding study, the primary aims of which were to evaluate the effects of glycemic load on chronic disease susceptibility biomarkers, e.g., markers of systemic inflammation, insulin resistance, and adipokines [3,4,5,6]. Participants were block-randomized based on body mass index and sex to receive two eucalorically similar controlled diets in a computer-generated, randomly assigned order for 28 days, with a 28 day washout period in between diets where participants could eat as desired. The study protocol, including the present ancillary study, was conducted in accordance with the ethical standards of the Declaration of Helsinki and approved by the Fred Hutch Institutional Review Board (8 March 2006 and 1 February 2008). All participants gave written informed consent. This trial was registered at clinicaltrials.gov as NCT00622661 (25 February 2008). This was a randomized trial. The randomization was on the order of the diets given to the participants.

### 2.2. Participants

Details on recruitment and study design have been published previously [3]. Briefly, healthy, non-smoking individuals between the ages of 18–45 years were recruited from the Greater Seattle area. Exclusion criteria comprised impaired fasting glucose measured at a study clinic visit (fasting blood glucose ≥ 5.6 mmol/L), any physician diagnosed condition requiring dietary modification or diet therapy, food allergies, regular use of hormones or anti-inflammatory medication, current pregnancy or plans to become pregnant, lactation, or heavy use of alcohol (>2 drinks/d). For the microbiome analyses specifically, if participants had taken antibiotics within 6 months of entering the intervention, their samples were excluded from the microbiome sample analysis. Of the 84 participants randomized, 80 completed all study activities and had complete biospecimen data. For this methods development study, we used a subset of nine randomly selected individuals.

### 2.3. Study Diets

Participants received two controlled diets: a low glycemic load diet characterized by whole grains, legumes, fruits, vegetables, nuts, and seeds (WG) or a high glycemic load diet, which was high in refined grains and added sugars (RG). All food was provided by the Fred Hutch Human Nutrition Laboratory during each intervention period. The two diets were designed to be comparable in energy and macronutrient distribution (15% energy from protein, 30% energy from fat, and 55% energy from carbohydrate) with diets differing in glycemic load (125 versus 250) and fiber (55 and 28 g/d) for the WG and RG diets, respectively. More details on the diet composition have been published elsewhere [3]. 

### 2.4. Sample Collection and Processing

Stool samples were collected at home using a collection tube containing 5 mL RNAlater^®^ (ThermoFisher, Waltham, MA, USA) and sterile 3 mm glass beads (ThermoFisher, Waltham, MA, USA) to facilitate sample dispersion in RNAlater^®^ [7,8]. Participants kept their samples in their home freezers and brought them to the study clinic on the last day of each diet period. Stool samples were stored in RNAlater^®^ at −80 °C at Fred Hutch. Stool samples were thawed and homogenized and aliquoted for analysis. One aliquot from the end of each intervention period from 9 participants was used for this analysis. 

### 2.5. Protein Extraction and Sample Preparation 

Each fecal sample (~0.4 g) was homogenized in 200 µL washing buffer (50 mM sodium phosphate pH 8, 0.1% Tween 20) with the addition of protease inhibitor cocktail (ThermoFisher, Waltham, MA, USA). The sample was vortexed and rotated at room temperature for 20 min on a rotating platform shaker. The homogenate was centrifuged at 4 °C and 200× *g* for 15 min. The aqueous supernatant was collected and kept on ice. The pelleted insoluble materials were resuspended in 200 µL washing buffer, and the same washing process was repeated 4 times. The aqueous supernatants from each wash were collected and combined. The sample was added to a lysis buffer to reach a final concentration of 6 M guanidine, 10 mM DTT, and 1 × protease inhibitor. The sample was vortexed and then centrifuged at 4 °C and 200× *g* for 15 min to collect the supernatant. About 50 µL cold acid washed glass beads (Sigma-Aldrich, St. Louis, MO, USA) was added to the supernatant. The sample was vortexed for 15 s 3 times with 30 s incubation on ice between each vortexing. After incubating the sample on ice for an additional 10 min, the sample was centrifuged at 4 °C and 14,000× *g* for 15 min. The aqueous supernatant was collected and the protein concentration was measured with Bradford assay. 

For each sample, 200 µg protein was used for proteomic analysis. The sample was incubated with 10 mM DTT at 50 °C for 1 h. Iodoacetamide (IDA) was added to the sample with a final concentration of 25 mM, and the sample was incubated at room temperature in the dark for 30 min. The proteins were precipitated by adding ¼ volume of 100% trichloroacetic acid (TCA) and incubating the sample on ice for 10 min. The sample was centrifuged at 4 °C and 14,000× *g* for 5 min. The protein pellet was rinsed twice with 200 µL ice-cold 90% acetone and air-dried before resuspension in 50 mM ammonium bicarbonate. The proteins were digested with MS grade trypsin (Thermo Fisher Scientific, Waltham, MA, USA) at a 1:30 enzyme to protein weight ratio in a two-step process. In the first step, half of the trypsin was added and the sample was incubated for 2 h at 37 °C and vortexed every 30 min. Then, the remaining trypsin was added and the mixture was incubated for an additional 16 h at 37 °C. The digested sample was dried down and re-suspended in 0.1% formic acid with a final concentration of 1 µg/µL for LC MS/MS analysis.

### 2.6. LC MS/MS Analysis 

The samples were analyzed by a Q ExactiveTM plus mass spectrometer (ThermoFisher) coupled with a nanoACQUITY HPLC system (Waters, Milford, MA, USA). One microgram of peptides were first loaded onto a trapping column (100 µm × 3 cm) and then separated with an analytical column (75 µm × 30 cm). The trapping column and the analytical column were packed with ProntoSIL 5 µm/120 Å C18 AQ beads (Mac-Mod, Chadds Ford, PA, USA). The analytical column was house-made with an emitter tip pulled with a Laser Fiber Puller P-2000 (Sutter Instruments, Novato, CA, USA) at the end of the column. The sample was loaded onto the trapping column with 98% Buffer A (0.1% formic acid in water) and 2% Buffer B (0.1% formic acid in acetonitrile) at a flow rate of 2 µL/min for 10 min and separated by the analytical column using a linear gradient from 5% to 30% B for 90 min, followed by flushing with 80% B for 10 min and equilibration with 2% B for 20 min. The LC gradient lasted 120 min with a flow rate of 0.3 µL/min. Electrospray ionization was operated in a positive mode at a voltage of 2.1 kV. The mass spectrometric analysis was performed using data dependent acquisition (DDA) with a mass range of 400 to 1200 *m*/*z*. The survey scan was acquired with 70,000 resolution at 200 *m*/*z* with an AGC target of 1e6 and max injection time of 100 ms. The precursors were isolated in the quadrupole within an isolation window of 1.6 *m*/*z*. The top 50 monoisotopic masses with 2 to 4 plus charges were selected with a minimum intensity threshold of 5e4 and were then fragmented by higher energy collisional dissociation (HCD) of 28 eV. 

### 2.7. Database Search

The MS/MS data were converted to mzML format and searched against the Uniprot human protein database (2015-07-23) for human host protein identification and the human gut microbiome database developed by the Human Microbiome Project (HMP) for human gut bacteria identification, respectively. The HMP human gut microbiome database includes 457 bacterial species identified in human fecal samples by metagenomics [9,10]. The data sets were processed and searched using the Trans-Proteomic Pipeline (TPP v4.6) [11,12] with the Comet algorithm [13]. Carbamidomethylation of cysteine was set as a fixed modification and oxidation of methionine was set as a variable modification. A maximum of five modifications and two mis-cleavages with a precursor mass tolerance of 20 ppm were allowed in the search. The peptide assignment was validated with PeptideProphet [14] and a probability score in correspondence with a false discovery rate (FDR) of 1% was applied to filter the peptides. 

### 2.8. Human Protein Data Analysis

The Skyline software (v3.6) [15] was used for quantitative analysis of the human protein data. The peptide identifications from the TTP were imported into Skyline to build the spectral library. Quantification was made at the MS1 level using the sum of the first 3 monoisotopic peaks. A restriction of a 5 min retention time window of the MS/MS identifications was applied to enhance the accuracy of library matching for peptide identification. The abundance of each peptide was normalized to total ion current (TIC) and presented as ions per million (ipm) using the following formula: normalized intensity (ipm) = (peptide intensity/TIC) × 1,000,000. Human protein quantification was inferred by sum-of-peptide quantification.

### 2.9. Metaproteomic Data Analysis

The identified bacterial peptides were input into Unipept [16] for metaproteome data analysis. For taxa analysis, peptides were assigned to taxa with the lowest common ancestor (LCA) approach using NCBI Taxonomy (https://www.ncbi.nlm.nih.gov/guide/taxonomy/) cross-references from matched UniProtKB proteins (https://www.uniprot.org/) in Unipept. Functional analysis based on GO terms (http://geneontology.org/) and EC numbers (https://www.genome.jp/kegg/annotation/enzyme.html) of the identified microbial peptides were also carried out in Unipept. All the quantification was based on the total peptide counts in their respective category. For GO terms, the total peptide count from all the proteins in the same GO term was used for quantitative analysis. For EC enzymes, the total peptide count for each enzyme was used for quantitative analysis.

### 2.10. Bioinformatics and Statistical Analysis 

Unsupervised clustering was achieved using Morpheus from the Broad Institute (https://software.broadinstitute.org/morpheus). Functional annotation and Enrichment of human proteins were carried by The Database for Annotation, Visualization, and Integrated Discovery (DAVID) v6.8 [17,18].

All the statistical analyses were carried in GraphPad. T tests, using the two-stage linear step-up procedure and controlling for FDR < 10%, were used to compare the GO biological processes and EC enzymes between the two diets. Ratio paired t-tests were used to compare the paired two diets for each participant. All the error bars in the figures are the standard error of the mean (SEM).

## 3. Results

### 3.1. Assay Reproducibility

**Figure 1:**
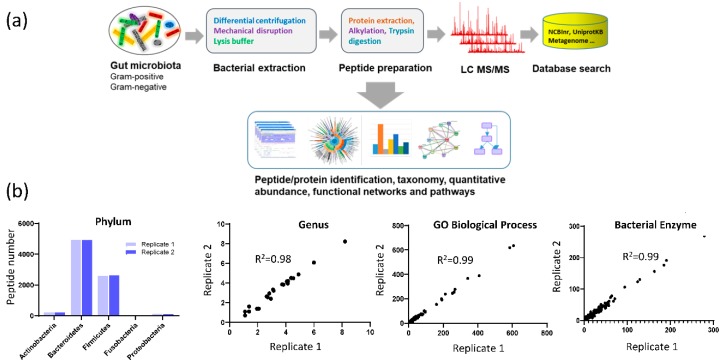
Study design and assay reproducibility. (**a**) metaproteomics approach; (**b**) reproducibility analysis based on replicate samples. Left panel showed the peptide number identified at the five major phyla. The right three panels were the correlation analyses of the replicates using the peptide numbers identified in Taxa, GO biological process, and bacterial enzymes, respectively.

The mass spectrometric data were searched against the HMP (Human Microbiome Project) database and Uniprot human protein database to identify microbial peptides and human derived peptides, respectively (Figure 1a). Using a threshold of 1% peptide level FDR, 47,353 unique microbial peptides and 5951 unique human peptides were identified in the fecal samples. On average, there were 7379 unique bacterial peptides and 1500 unique human peptides identified in each fecal sample. We further assessed the reproducibility of the study approach by analyzing sample replicates. As shown in Figure 1b, the data were highly reproducible in terms of the identification of bacteria at the phylum level, genus level, number of peptides assigned to GO biological processes, and microbial enzymes, with R^2^ ≥ 0.98 and *p* < 0.0001.

### 3.2. Human proteins identified in fecal samples

**Figure 2:**
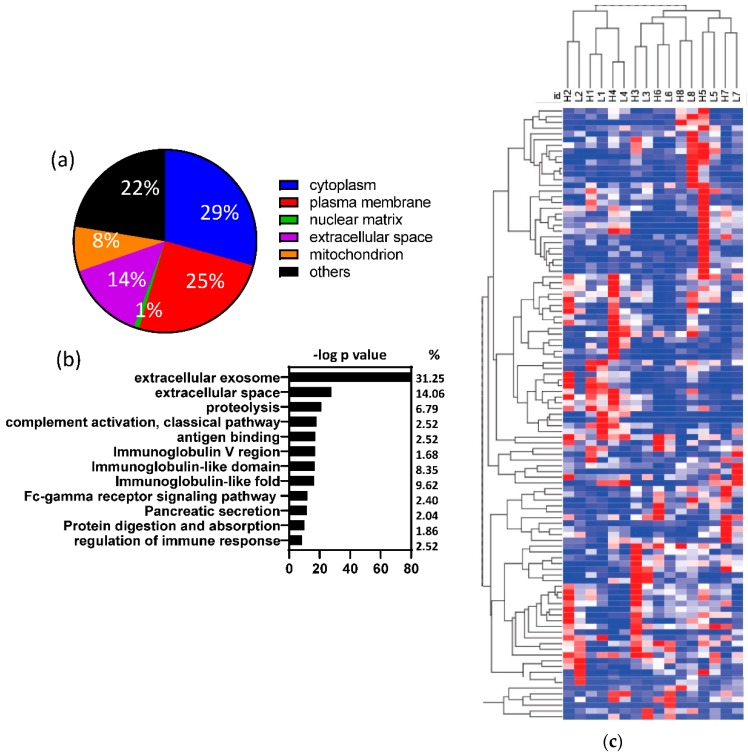
Fecal human proteins were not significantly different between the diets. (**a**) Cellular location of the fecal human proteins; (**b**) Enrichment analysis of the fecal human proteins; (**c**) Hierarchical cluster analysis of fecal human proteins. The same participant on different diets clustered together based on their fecal human proteins. The number denotes the participant number; H = high glycemic load, RG diet; L = low glycemic load, WG diet; ns: non-significant.

The human peptides identified in the fecal samples could be matched to 1695 human proteins or protein groups. As shown in Figure 2a, 14% of these proteins were secreted extracellular proteins. The majority of the human proteins (86%) were plasma membrane or intracellular proteins, which may be from human colon epithelial cell turnover or breakdown. Functional annotation further showed that the human proteins found in the fecal samples were highly enriched in three categories (Figure 2b): (1) extracellular destination (exosome, extracellular space, and pancreatic secretion); (2) proteolysis (including digestion and absorption); and (3) immune-related proteins. To determine if the human proteins present in fecal samples differed by diet, we compared the protein expressions between the two diets. Although the majority of the human proteins present in the fecal samples did not show significant differences between the two dietary treatments (Supplemental Table S1), alpha amylase (*p* < 0.000001) was higher in the RG diet and chymotrypsin was higher in the WG diet (*p* < 0.000001). Furthermore, the same participant on the different diets were completely clustered together by hierarchical clustering of human-derived analysis (Figure 2c). 

### 3.3. Fecal Microbial Composition Based on Metaproteomics Analysis 

**Figure 3:**
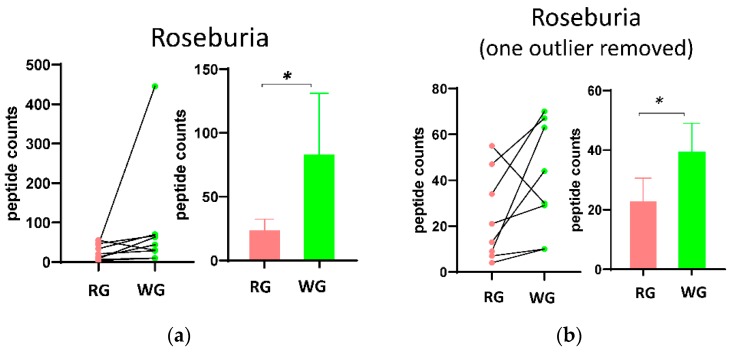
Changes in the composition and relative abundance of the stool microbiome. (**a**,**b**) Selected taxa differences between the two dietary treatments (RG and WG). paired *t* test: * *p* < 0.05.

To evaluate potential effects of the diets on microbiome composition, we used Unipept to assign taxa for the microbial peptides identified in the fecal samples. We identified 5 major phyla and 58 genera in the 9 participants. The microbial composition at the phylum level (Supplemental Figure S1a and S1b) showed Firmicutes was statistically significantly higher (1.2 fold, *p* = 0.037) in the WG diet, although other major phyla did not differ. At the genus level, Roseburia was significantly enriched (2.3 fold, *p* = 0.023) in the WG diet, but other genera were not. One of the participants showed an extreme enrichment in Roseburia in the WG diet. After removing that participant from the data, the increase of Roseburia was still statistically significant (Figure 3b). The taxa data can be found in Supplemental Table S2.

### 3.4. Diet-Induced Significant Changes in GO Biological Processes 

**Figure 4:**
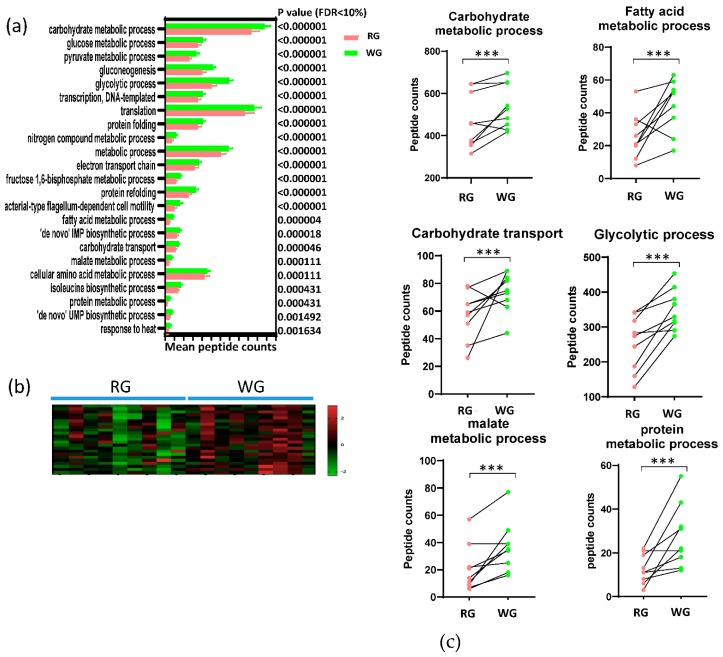
Significant differences in GO biological processes. (**a**) Biological processes that were significantly different between the two diets; (**b**) Heat map of the significant biological processes; (**c**) Selected biological processes. * *p* < 0.05, ** *p* < 0.01. *** *p* < 0.001.

There were 1073 GO terms in biological processes matched to the bacterial peptides identified in the 18 samples. We identified 23 GO biological processes that were significantly different between the two diets after FDR adjustment (Figure 4a). In fact, all the 23 biological processes were significantly enhanced in the WG diet (Figure 4b) and the majority (70%) of the processes were related to metabolic processes such as carbohydrate, fatty acid, and protein metabolism, and carbohydrate transport. Other significant biological processes included transcription, translation, and protein folding, all of which were related to the proliferation of microbes. The contrasts between RG and WG diets for each individual are shown in Figure 4c for selected biological processes. Pathways significantly higher after the WG diet included carbohydrate transport, glycolytic process, protein metabolic process, and malate metabolic process. Additionally, 78% and 89% of the participants showed higher carbohydrate metabolic processes and fatty acid metabolic processes after the WG diet, respectively.

### 3.5. WG Induced Significant Enrichment in Bacterial Enzymes Involved in the Production of SCFA and the Degradation of Fatty Acids

**Figure 5:**
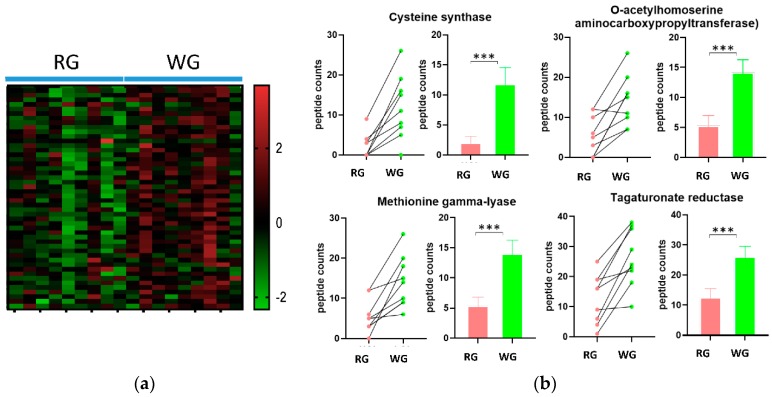
Significant differences in GO biological processes by diet. (**a**) Heatmap of the enzymes; (**b**) The top four enzymes with the highest fold change by diet. *** *p* < 0.001.

The GO biological process analysis for metabolic processes was higher in the WG diet, especially the carbohydrate metabolism. To further investigate microbial metabolism, we evaluated the bacterial enzymes affected by the diets. There were 1031 bacterial enzymes (according to EC numbering systems) matched to the bacterial peptides identified in the 18 samples. Using *t*-tests adjusted using FDR (<10%), we identified 48 bacterial enzymes that were significantly different between the diets (Table 1). Most of these significantly different enzymes showed greater abundance in the WG diet compared to the RG diet (Figure 5a,b, Supplemental Figure S2). We then mapped these enzymes to KEGG pathways and found that the majority of these enzymes were implicated in the pathways of biosynthesis of secondary metabolites, carbon fixation, and carbohydrate and lipid metabolism (Supplemental Table S3). Among these pathways, starch, pyruvate, sucrose metabolism, and glycolysis were all higher in the WG diet. In contrast, the abundances of three bacterial enzymes (NADH peroxidase, beta-galactosidase, and endo-alpha-*N*-acetylgalactosaminidase), which are relevant to oxidative stress and mucin degradation, were significantly higher following the RG diet (Table 1). 

**Figure 6:**
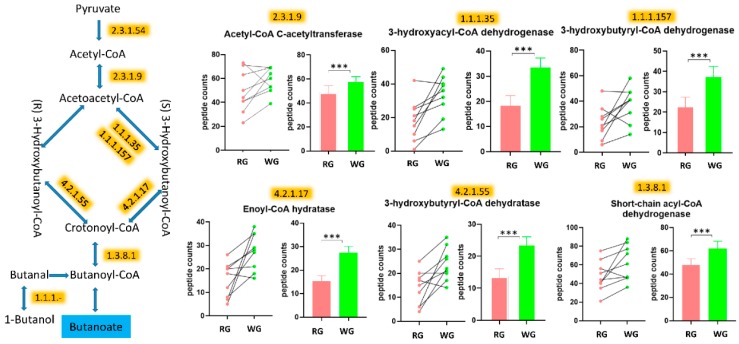
The WG diet led to enhanced metabolism of butyrate. The enzymes (highlighted) were significantly higher following the WG diet. The drawing of the pathways was adapted from KEGG pathway databases. Some steps from the original pathways are not presented in the figures. *** *p* < 0.001.

**Figure 7:**
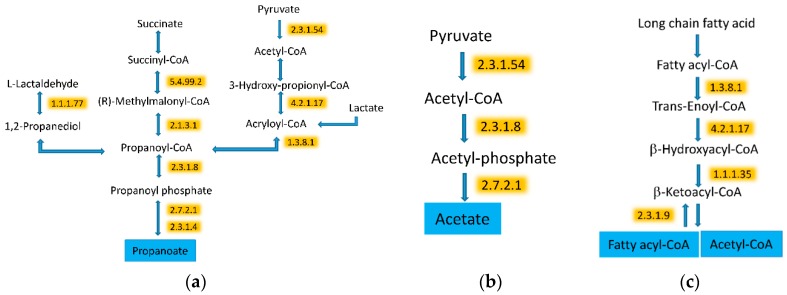
The WG diet induced metabolism of SCFA and metabolism of fatty acids. (**a**) Propanoate; (**b**) Acetate; (**c**) Fatty acid. All of the highlighted enzymes were increased by the WG diet. The drawing of the pathways was adapted from KEGG pathway databases. Some steps from the original pathways are not presented in the figures.

We observed that the fermentation pathways of three SCFAs, acetate, propanoate, and butanoate (e.g., butyrate) were all higher after the WG diet. Specifically, eight enzymes related to the butyrate pathways were significantly higher after the WG diet as compared to the RG diet (Figure 6 and Table 1). Two enzymes, phosphate acetyltransferase and acetate kinase, involved in the synthesis of acetate were also higher after the WG diet (Figure 7). Furthermore, nine enzymes related to the three propanoate synthesis pathways were also higher after the WG diet. 

In addition to the changes related to the higher carbohydrate metabolism, we also observed protein changes indicating higher degradation and synthesis of fatty acids. Three enzymes involved in the degradation of fatty acids were increased by the WG diet (Figure 7c). However, acetyl-CoA C-acetyltransferase, which leads to fatty acid synthesis, was also higher in the WG diet.

## 4. Discussion

Using a metaproteomic approach, we demonstrated that the fecal microbiome responded to dietary patterns. The number of different kinds of bacterial enzymes was higher in the WG diet than in the RG diet. The microbiome in the WG diet had significantly higher concentrations of enzymes associated with fiber fermentation and fatty acid metabolism. In contrast, the microbiome produced mucin degrading enzyme in response to the RG diet.

Pancreatic secretion of enzymes that aid in the digestion of diets are responsive to changes in the composition of dietary intake [19,20,21]. In our study, alpha amylase was higher in the RG diet and chymotrypsin was higher in the WG diet. Chymotrypsin and amylase are enzymes released from the pancreas during normal digestion and are often found in stools. Dietary fibers with varying complexity impact pancreatic enzymes differently [22]. Pectin, a fiber found in fruits, has been shown to increase chymotrypsin, whereas wheat and oat brans have been shown to decrease amylase activity [19]. Pectin has been shown to reduce the activity of amylase in duodenal fluid in vitro [23]. Furthermore, non-soluble dietary fibers may have direct molecular interaction and adsorb enzymes which also alters pancreatic enzyme activity [19,24]. 

The host exposure to microbial metabolites varies based upon the chemical structure of the dietary components and the suite of microbial enzymes present in the gut [25,26,27,28,29,30,31,32,33,34,35,36,37,38]. Microbial metabolism of complex dietary polysaccharides relies on specific microbial pathways and enzymes [39]. Our results show that the microbiome in the WG diet enriched in dietary fiber and whole grains had enzymes associated with the degradation of oligosaccharides, such as pectin. Pectin is comprised of units of d-galacturonate and bacteria use d-galacturonate as an energy source [40]. We identified two enzymes in the conversion of d-galacturonate to pyruvate, d-tagaturonate reductase (EC 1.1.1.5) and enoyl-CoA hydratase (EC 4.2.1.17; altronate dehydratase), which were significantly enriched in the WG diet [41,42]. 

SCFAs are important in gut homeostasis [43], regulation of inflammatory responses [44], immune response, gut hormone secretion [45], and can induce differentiation and apoptosis [46,47,48] in the host [43,44,45,49,50,51]. Bacteria ferment fiber to SCFAs, predominantly acetate, butyrate, and propionate [52,53,54,55,56]. We found that enzymes involved in acetate, butyrate, and propanoate production were higher in the WG diet. Acetate is used in both host and microbial metabolism. In the host, acetate is used as an energy source for liver and peripheral tissues and acts as a signaling molecule in gluconeogenesis and lipogenesis [57]. Microbial long chain fatty acid metabolism was also higher in the WG diet, likely through the increased flux of acetate from fermentation of fiber. Others have shown similar increased metaproteomic signal in fatty acid metabolism with starch enrichment [1,58]. Butyrate is produced via multiple pathways including the acetyl-CoA [59,60,61], glutarate [62,63,64], 4-aminobutyrate [65,66,67], and lysine pathways [68,69,70]. The dominant pathway associated with butyrate production is the acetyl-CoA pathway, which was higher in the microbiome in the WG enriched diet [57,59,71,72,73,74]. We found that *Roseburia*, a genus that produces butyrate by condensing two moles of acetate, was significantly higher in the WG diet. Butyrate serves as the main energy source for colonocytes and protects against inflammation [57]. It also affects the regulation of apoptosis and cellular proliferation, which may result in a reduced risk of colon cancer [48]. Enzymes in the propanoate pathway, a major fiber fermentation product formed through the microbial succinate and acrylate pathways [75,76,77,78], was also enriched in the WG diet. Propanoate serves as a precursor for gluconeogenesis and reduces the synthesis of hepatic cholesterol [79]. Propanoate was also associated with a decrease in insulin secretion in pancreatic islet cells of rats [80].

Mucins on gut epithelial cells form a barrier between the gut microbiome and their hosts [58]. Our data suggests that consumption of a refined grain diet promotes the expansion and activity of colonic mucus-degrading bacteria [1,81]. There was enrichment in the repertoire of glycosyl hydrolases (GH) associated with mucin degradation including *β*-galactosidases (GH2, GH20, and GH42) and endo-alpha-*N*-acetylgalactosaminidase (GH101) in the RG diet [81]. Our dietary intervention expands upon the existing literature and suggests that although the RG diet contained more dietary fiber than the average US diet (25 g versus 15 g), the composition of the dietary fiber in the RG diet may have promoted the expansion and activity of colonic mucin degrading bacteria. In addition, the RG diet was also associated with increased NADH peroxidase. Peroxidases reduce toxic H_2_O_2_ often generated from oxygen at the oxic/anoxic interface of the intestinal epithelium by facultative bacteria [82,83,84]. Degradation of host-derived glycans has major implications for inflammation-associated colitis, colorectal cancer, and susceptibility to infection [58,85,86,87,88].

We have shown that the interrogation of the metaproteome at a functional level could reflect reasonable metabolic shifts in the microbial response to a dietary intervention of differing dietary patterns. The process of reliable annotation of protein function from a gene sequence is critical for the effective use of metaproteomic data from the human microbiota [89]. Efforts, such as the HMP studies [90], have been made to overcome the hurdles in metaproteomic database searches. Currently, we are limited by the number of bacterial genomes available in the reference databases. We anticipate that as sequencing technologies advance, using longer read nucleic acid sequencing, we will be able to better identify and curate the novel proteins from the genes in genomes of yet-to-be cultured bacteria. Although we used a small subset of samples available from a dietary intervention as a feasibility and proof-of-concept study, we have developed and optimized the methodology and shown a biologically plausible response of the human microbiome to the dietary intervention.

## 5. Conclusions

We presented a proof-of-concept analysis that showed differences in the metaproteome that varied with dietary pattern under controlled experimental dietary conditions. The microbiome has a major role in the production of compounds in the gut lumen and these microbial metabolites may influence human health. Future work should include kinetic characterization of putative mucin- and fiber-degrading enzymes and measurement of the fecal metabolome to understand the dynamics and specificity that impact the exposure of microbial metabolites on the human host.

## Figures and Tables

**Table 1:** Bacterial enzymes with significantly different abundance between the two diets.

		PEPTIDE COUNTS Average (SEM)		
EC number	Enzyme Name	RG	WG	*p* value*
**Higher after WG Diet**				
1.1.1.-	Oxidoreductases Acting on the CH-OH group of donors with NAD(+) or NADP(+) as acceptor	17.2 (4)	25.2 (5.4)	0.002584
1.1.1.1	Alcohol dehydrogenase	25.9 (11.8)	35.1 (9.6)	0.000514
1.1.1.157	3-hydroxybutyryl-CoA dehydrogenase	23.1 (12.8)	37.9 (13.7)	<0.000001
1.1.1.35	3-hydroxyacyl-CoA dehydrogenase	18.1 (12.4)	33.3 (11.7)	<0.000001
1.1.1.37	Malate dehydrogenase	21.4 (14.2)	39 (11.9)	<0.000001
1.1.1.58	Tagaturonate reductase	12.7 (8.3)	26.3 (9.5)	<0.000001
1.1.1.69	Gluconate 5-dehydrogenase	14.2 (7.3)	23.9 (6.8)	0.000272
1.1.1.86	Ketol-acid reductoisomerase (NADP(+))	52.3 (19.3)	60.8 (13.4)	0.001469
1.17.7.4	4-hydroxy-3-methylbut-2-enyl diphosphate reductase	21.7 (10.6)	30.8 (7.2)	0.0006
1.2.1.-	Oxidoreductases Acting on the aldehyde or oxo group of donors with NAD(+) or NADP(+) as acceptor	189.1 (51.9)	214.2 (44.2)	<0.000001
1.2.7.-	Oxidoreductases Acting on the aldehyde or oxo group of donors with an iron-sulfur protein as acceptor	159.2 (64.8)	185.8 (37.2)	<0.000001
1.3.8.1	Butyryl-CoA dehydrogenase	47.7 (16.4)	61.9 (19)	<0.000001
2.2.1.1	Transketolase	22.3 (13.1)	36.4 (13.1)	<0.000001
2.2.1.2	Transaldolase	20.8 (7.1)	29.2 (13.7)	0.001469
2.3.1.54	Formate C-acetyltransferase	36.1 (23.3)	44 (24.1)	0.002964
2.3.1.8	Phosphate acetyltransferase	17.3 (11.4)	28.3 (8.8)	0.000034
2.3.1.9	Acetyl-CoA C-acetyltransferase	48.7 (17.2)	58.4 (10.5)	0.000231
2.4.1.1	Glycogen phosphorylase	37.6 (13.6)	48.4 (14.6)	0.000041
2.5.1.47	Cysteine synthase	2.1 (3.1)	11.9 (7.9)	0.000231
2.5.1.49	O-acetylhomoserine aminocarboxypropyltransferase	5.3 (5)	14.2 (6.2)	0.000814
2.6.1.52	Phosphoserine transaminase	22.2 (5.9)	30.3 (7.9)	0.00225
2.7.1.92	5-dehydro-2-deoxygluconokinase	16.8 (9.1)	24.6 (7.1)	0.003394
2.7.2.1	Acetate kinase	27.8 (9.2)	37.4 (9.6)	0.000272
2.7.2.3	Phosphoglycerate kinase	75.3 (30.6)	107.3 (23.7)	<0.000001
2.7.7.27	Glucose-1-phosphate adenylyltransferase	22.9 (15.6)	31.1 (10.2)	0.001955
2.7.7.6	DNA-directed RNA polymerase	124.1 (30.9)	136.3 (23.6)	0.000004
2.7.7.8	Polyribonucleotide nucleotidyltransferase	28.8 (14.1)	36.8 (11.6)	0.002584
2.7.9.1	Pyruvate, phosphate dikinase	130.9 (35.2)	168.9 (40.3)	<0.000001
3.5.-.-	Hydrolases Acting on carbon-nitrogen bonds, other than peptide bonds	6.6 (6.2)	15.1 (11.3)	0.001271
3.6.3.14	H(+)-transporting two-sector ATPase	33.8 (13.4)	47.1 (16.4)	<0.000001
3.6.5.3	Protein-synthesizing GTPase	93.8 (25.9)	112.7 (30.6)	<0.000001
4.1.1.49	Phosphoenolpyruvate carboxykinase (ATP)	126.1 (50.6)	157 (26.1)	<0.000001
4.1.2.13	Fructose-bisphosphate aldolase	65.2 (25.3)	92.8 (20.9)	<0.000001
4.2.1.11	Phosphopyruvate hydratase	24.7 (13.6)	32.9 (14.8)	0.001955
4.2.1.17	Enoyl-CoA hydratase (altronate dehydratase)	15.2 (7.4)	27.4 (7.8)	0.000004
4.2.1.55	3-hydroxybutyryl-CoA dehydratase	13.7 (7.2)	23.8 (6.9)	0.00014
4.4.1.11	Methionine gamma-lyase	5.4 (4.1)	14.1 (6.4)	0.001097
5.3.1.1	Triose-phosphate isomerase	43.2 (15)	56.7 (12.2)	<0.000001
5.3.1.9	Glucose-6-phosphate isomerase	29.1 (11.8)	42.1 (11.6)	<0.000001
5.4.2.2	Phosphoglucomutase (alpha-d-glucose-1,6-bisphosphate-dependent)	33.4 (15.7)	50.7 (19.3)	<0.000001
5.4.2.8	Phosphomannomutase	13.7 (8.1)	22.4 (9.6)	0.000946
5.4.99.2	Methylmalonyl-CoA mutase	20.8 (15.4)	28.6 (15.5)	0.003394
6.3.1.2	Glutamine synthetase	24.9 (18.7)	37.3 (20.5)	0.000003
6.3.2.6	Phosphoribosylaminoimidazolesuccinocarboxamide synthase purine	20.8 (7)	29.4 (11.2)	0.001097
6.3.5.5	Carbamoyl-phosphate synthase (glutamine-hydrolyzing)	22.2 (9.5)	36.4 (7.2)	<0.000001
**Higher after RG Diet**				
1.11.1.1	NADH peroxidase	72.8 (29.4)	57.8 (20.1)	<0.000001
3.2.1.23	Beta-galactosidase	46.3 (21.1)	37.9 (14.4)	0.001469
3.2.1.97	Endo-alpha-*N*-acetylgalactosaminidase	21.2 (20.8)	7.4 (6.2)	<0.000001

*<FDR 10%.

## Supplementary Materials

Supplementary materials can be found at https://www.mdpi.com/2076-2607/8/3/379/s1. Supplemental Table S1: Comparison of human proteins by the two diets; Supplemental Table S2: Identification of bacterial taxa based on metaproteomics analysis; Supplemental Table S3: Mapping of the microbiome enzymes significantly affected by the diets to the KEGG pathways; Supplemental Figure S1: Composition and relative abundance of (a) bacterial phyla and (b) changes in Firmicutes in the metaproteomic data by diet (RG and WG diets); Supplemental Figure S2: Significant differences in GO biological processes by diet.
